# Ultrasound-Assisted Preparation of Hyaluronic Acid-Based Nanocapsules with an Oil Core

**DOI:** 10.3390/ma17184524

**Published:** 2024-09-14

**Authors:** Natan Rajtar, Grzegorz Łazarski, Aleksander Foryś, Łukasz Otulakowski, Barbara Trzebicka, Dorota Jamróz, Mariusz Kepczynski

**Affiliations:** 1 Faculty of Chemistry, Jagiellonian University, Gronostajowa 2, 30-386 Kraków, Poland; natan.rajtar@doctoral.uj.edu.pl (N.R.); grzegorz.lazarski@doctoral.uj.edu.pl (G.Ł.); dorota.jamroz@uj.edu.pl (D.J.); 2 Doctoral School of Exact and Natural Sciences, Jagiellonian University, Prof. S. Łojasiewicza 11, 30-348 Krakow, Poland; 3 Centre of Polymer and Carbon Materials, Polish Academy of Sciences, 41-819 Zabrze, Poland; aforys@cmpw-pan.pl (A.F.); lotulakowski@cmpw-pan.pl (Ł.O.); btrzebicka@cmpw-pan.pl (B.T.)

**Keywords:** hyaluronic acid, nanocapsules, nanoemulsion, molecular dynamics simulations, sonication, drug carriers

## Abstract

Liquid-core nanocapsules (NCs) coated with amphiphilic hyaluronic acid (AmHA) have been proposed for the preparation of drug and food formulations. Herein, we focused on the use of ultrasound techniques to (i) optimize the polysaccharide chain length with respect to the properties of NCs stabilized with AmHAs and (ii) form oil-core nanocapsules with a coating composed of AmHAs. The results indicate that sonication is a convenient and effective method that allows for a controlled reduction in HA molecular weight. The initial (H-HA) and degraded (L-HA) polysaccharides were then reacted with dodecylamine to obtain hydrophobic HA derivatives (HA-C12s). Then, NCs were prepared based on HA-C12s using ultrasound-assisted emulsification of glyceryl triacetate oil. The nanocapsules coated with L-HA-C12 showed greater stability compared to the longer-chain polysaccharide. Molecular dynamics (MD) simulations revealed that HA-C12 readily adsorbs at the water–oil interphase, adopting a more compact conformation compared to that in the aqueous phase. The dodecyl groups are immersed in the oil droplet, while the main polysaccharide chain remaining in the aqueous phase forms hydrogen bonds or water bridges with the polar part of the triglycerides, thus increasing the stability of the NC. Our research underscores the usefulness of ultrasound technology in preparing suitable formulations of bioactive substances.

## 1. Introduction

Nanomedicine deals with the application of nanotechnology in medicine, including the development of nanometric carriers for drugs (drug delivery systems, DDSs) and diagnostic contrast agents [1]. Over the past few decades, various nanomedicines have been approved for clinical use by the U.S. Food and Drug Administration (FDA) or are undergoing clinical trials [2]. Properly designed polymer-based DDSs can act as drug reservoirs (i.e., enable prolonged and controlled release of drugs) and can deliver active substances to a designated site in a living organism in a controlled manner (targeted therapy) [3]. In recent years, nanocapsules (NCs) with a liquid core (nanoemulsions), consisting of an oily core surrounded by a polymeric shell, have gained great importance in DDS development due to their versatile structures and tunable physicochemical properties [4]. The oil core enables effective encapsulation of hydrophobic substances (such as drugs, dietary supplements, or nutrients), while the polymer envelope imparts desired pharmaceutical properties to the carrier, such as drug protection, prolonged stability, and targeted delivery. To date, plant oils (such as corn oil, linseed oil, olive oil, peanut oil, sesame oil, soybean oil, or mixtures thereof), fish oil, oleic acid [5], and caprylic/capric triglyceride (Miglyol 812) [6] have been most commonly used as the oil phase in nanoemulsion formulations. Some properties of the oil phase were shown to affect the size and stability of NCs [4].

Amphiphilic derivatives of hyaluronic acid (AmHAs) have been proposed as effective polymer shells for coating NCs with a liquid core. HA belongs to the glycosaminoglycans (GAGs) found in the extracellular matrix (ECM) of living organisms. This polysaccharide has some unique and superior biological properties, such as biodegradability, biocompatibility, non-toxicity, and non-immunogenicity. In addition, it can bind to the CD44 receptor, a protein that is overexpressed in cancer cells [7], which is desirable for DDSs targeting anticancer drug delivery. AmHAs are obtained by attaching hydrophobic groups along the polysaccharide chain. The hydrophobic part of the AmHA macromolecule anchors at the water–oil interface, while the hydrophilic part, which is highly hydrated, remains in the aqueous phase. A number of hydrophobically modified hyaluronate derivatives, differing in the degree of substitution (DS) and length of hydrophobic moieties have been synthesized and used to stabilize nanoemulsions. For example, HA (*M*_w_ = 11.6 kDa) conjugated to a polyethylene glycol/poly(ε-caprolactone) copolymer was used to prepare oil-core NCs via an oil-in-water emulsion solvent diffusion method, which required the application of an organic solvent (ethyl acetate in this case) [8]. Cadete et al. modified HA with *M*_w_ = 200,000 Da by grafting with dodecyl groups (DS = 2.5% to 5.0%) and used it to cover caprylic/capric triglyceride nanodroplets that were additionally stabilized with the surfactant Tween 80 [5]. However, the application of surfactants is often detrimental to the safety and bioavailability of nanoemulsions [9]. Meanwhile, another study showed that the use of alkyl derivatives of HA (differing in the degree of substitution and length of hydrophobic units) to stabilize oleic acid nanodroplets obtained by sono-assisted emulsification did not require the use of surfactants [10]. The stability of the prepared nanoemulsions was attributed to spatial repulsion between the shell-forming HA derivatives, which were negatively charged. In further work, Bednorz et al. used HA (*M*_w_ = 150,000 g/mol) grafted with dodecylamine to stabilize the nanoemulsion of various edible oils and their mixtures [4].

The effect of HA molecular weight on the properties of HA-based capsules has not been systematically addressed, but it would be worth investigating since the bioactivity of this polysaccharide may depend on it [11]. Depending on its molecular weight, HA can play different biological functions. In mammals, HA exists in a high-molecular-weight (H-HA) form with a mass in the range of 10^6^–10^7^ Da [12]. H-HA is generally assumed to exhibit anti-inflammatory, immunosuppressive, and anti-angiogenic properties, while low-molecular-weight (less than 100 kDa) HA (L-HA) has pro-inflammatory, angiogenic, and immunostimulatory effects [13,14].

In this study, we focused on the use of ultrasound techniques to (i) optimize the polysaccharide chain length with respect to the properties of NCs stabilized with amphiphilic HA derivatives (AmHAs), and (ii) form polymer nanocapsules with an oil core and shell composed of AmHAs. For this purpose, commercially available high-molecular-weight HA (H-HA) was degraded using ultrasound. The initial (H-HA) and degraded polymer (L-HA) was then reacted with dodecylamine, resulting in amphiphilic HA derivatives (HA-C12s). The HA-C12s were used to produce polymer aggregates and oil-core nanocapsules. In both cases, ultrasound was used, which made it much easier to obtain the desired objects and provided greater uniformity of size. The sizes and morphology of HA-C12-based NCs were characterized by light scattering and cryo-transmission electron microscopy (cryo-TEM) methods. Comparison of the results for H-HA-C12 and L-HA-C12 made it possible to assess the effect of polysaccharide chain length on the properties and stability of the obtained nanoemulsions. In addition, computer simulations were performed to reveal the conformation of HA-C12 chains at the water–oil interface. It is worth noting that the ionic strength of the environment has a significant effect on the behavior of HA chains, which are polyelectrolytes. Atomic force microscopy studies have shown that increasing the salt concentration changes the conformation and nanomechanical properties of this polysaccharide [15]. HA macromolecules in electrolyte solutions become more flexible and assume more compact conformations than in pure water. Therefore, we performed both experimental studies and computer simulations at physiological salt concentrations. Our research underscores the usefulness of ultrasound technology in preparing appropriate carriers that can protect cargo from degradation and improve its adsorption in the human body.

## 2. Materials and Methods

### 2.1. Materials

Hyaluronic acid sodium salt from Streptococcus equi (H-HA, molecular weight of 1500–1800 kDa, ≤1% protein, CAS number 9067-32-7), dodecylamine (≥99%, CAS number 124-22-1), *N*-(3-dimethylaminopropyl)-*N*′-ethylcarbodiimide (EDC, CAS number 25952-53-8), *N*-hydroxysuccinimide (NHS, CAS number 6066-82-6), *N*,*N*-dimethylformamide (DMF, CAS number 68-12-2), *N*-methylformamide (NMF, CAS number 123-39-7), phosphate-buffered saline (PBS, tablets), and glyceryl trioctanoate (GTO, density of 0.956 g/mL at 20 °C, CAS number 538-23-8) were purchased from Sigma Aldrich (St. Louis, MO, USA). 1,6-Diphenyl-1,3,5-hexatriene (DPH, CAS number 1720-32-7) was obtained from Fluka (Steinheim, Germany). Dowex 50WX8 ion exchange resin (CAS number 69011-20-7) was purchased from Merck Life Science (Darmstadt, Germany). Poly(ethylene oxide) standards with molecular weights of 26.1–969 kDa were obtained from PSS-Polymer Standards Service Inc. (Amherst, MA, USA). 

### 2.2. Degradation of Low Molecular Weight (H-HA)

Low-molecular-weight hyaluronic acid (L-HA) was prepared by degradation of commercial H-HA using ultrasonication. The degradation process of the sample placed in an ice bath was carried out using a VCX 500 sonicator (Sonics & Materials. Inc., Newtown, CT, USA). Two types of samples differing in volume and concentration of H-HA were sonicated:

Sample 1. A solution of H-HA (4 mL, 1 g/L) in deionized water was stirred overnight until the polymer was completely dissolved. The solution was sonicated for 10 min (power—9 W, pulse duration—2 s, break duration—2 s). The sonication was repeated five times, each time shaking the mixture beforehand. In the sixth cycle, the power of the device was raised to 100 W. Each time, 100 μL of the solution was taken for chromatographic analysis. The sample was passed through a 0.45 μm filter to remove any contamination from the sonicator tip.

Sample 2. A solution of H-HA (20 mL, 12.5 g/L) was prepared in deionized water as described above and sonicated for 4 × 15 min (power—300 W, pulse duration—2 s, break duration—2 s). 100 μL of the solution was passed through a 0.45 μm filter and used in chromatographic analysis.

### 2.3. Size Exclusion Chromatography (SEC)

SEC analyses were performed using an OMNISEC chromatograph (Malvern Instruments Ltd., Malvern, UK) equipped with a PolySep-SEC GFC-P Linear, LC Column 300 × 7.8 mm column (Phenomenex, Torrance, CA, USA). A 0.1 M NaNO_3_ solution was used as an eluent. The flow rate of the eluent was 0.8 mL/min. PEG standards of known molecular weight were used for calibration.

### 2.4. Dynamic Light Scattering (DLS)

DLS measurements were performed using a Brookhaven BI-200 goniometer (Brookhaven Instruments, Holtsville, NY, USA) with vertically polarized incident light at 632.8 nm (He-Ne laser, 35 mW) and equipped with a Brookhaven BI-9000 AT digital autocorrelator (Brookhaven Instruments, Holtsville, NY, USA). The intensity of scattered light was measured at an angle of 90°. To obtain relaxation rate (*Γ*) distributions, the autocorrelation functions were analyzed using the CONTIN method, which provided distributions of the apparent diffusion coefficient *D*, defined as follows:

*D* = *Γ*/*q*^2^, (1)

where *q* is the magnitude of the scattering vector, *q* = (4*πn*/*λ*)sin(*θ*/2), and *n* is the refractive index of the medium). The apparent hydrodynamic diameter ($D_{h}^{90}$) was calculated using the Stokes–Einstein equation:

(2) $$D_{h}^{90}=\frac{kT}{6\pi \eta D},$$

where *k* is the Boltzmann constant, and *η* is the viscosity of water at temperature *T*. The dispersity of particle sizes was given as $\mu_{2}/{\bar{\Gamma }}^{2}$, where $\bar{\Gamma }$ is the average relaxation rate, and $\mu_{2}$ is its second moment. Measurements were made for different concentrations of L-HA-C12 and H-HA-C12-based nanocapsules (NCs) at 20 °C.

### 2.5. Zeta Potential Measurements

ζ-potential measurements were performed using a Zetasizer Ultra apparatus (Malvern Instrument Ltd., Malvern, UK). The time-dependent autocorrelation function of the photocurrent was acquired every 10 s with 15 acquisitions for each run at 25 °C.

### 2.6. Turbidimetric Measurements

Stability measurements of the nanocapsule dispersions were carried out using a Turbiscan optical analyzer (TurbiSoft Classic 2.2.0.101, Formulaction, Toulouse, France). The dispersion (polymer concentration of 1 mg/mL) was placed in a glass tube (10 cm in length and 1.5 cm in diameter) and illuminated with a near-infrared light beam (λ = 850 nm). Changes in backscattered light by the sample over time were recorded in the three equal parts: the lower, middle, and upper parts of the sample. Liquid column height counted from the bottom of a glass tube was 57 mm (19 mm step) for L-HA and 60 mm (20 mm step) for H-HA. Data were collected every 3 h for 96 h.

### 2.7. Conventional (TEM) and Cryogenic Transmission Electron Microscopy (Cryo-TEM)

The morphology of objects suspended in phosphate-buffered saline (PBS) was observed by cryo-TEM. Details of sample preparation and measurements were described previously [16]. A Tecnai F20 X TWIN microscope (FEI Company, Hillsboro, OR, USA) equipped with a field emission gun operating at an acceleration voltage of 200 kV was used for visualization. Images were captured with a Gatan Rio 16 CMOS 4k camera (Gatan Inc., Pleasanton, CA, USA) and processed with Gatan Microscopy Suite (GMS, 3.31.2360.0) software (Gatan Inc., Pleasanton, CA, USA). The morphology of dried objects was observed by conventional TEM. Briefly, a dispersion of nanoparticles with a polymer concentration of 1 mg/mL was placed on a copper grid (Quantifoil R 2/2; Quantifoil Micro Tools GmbH, Großlöbichau, Germany) and negatively stained with UranyLess for 30 s, then dried at room temperature and observed under the Tecnai F20 X TWIN microscope (FEI Company, Hillsboro, OR, USA).

### 2.8. Synthesis of HA-C12s

**Figure 1:**
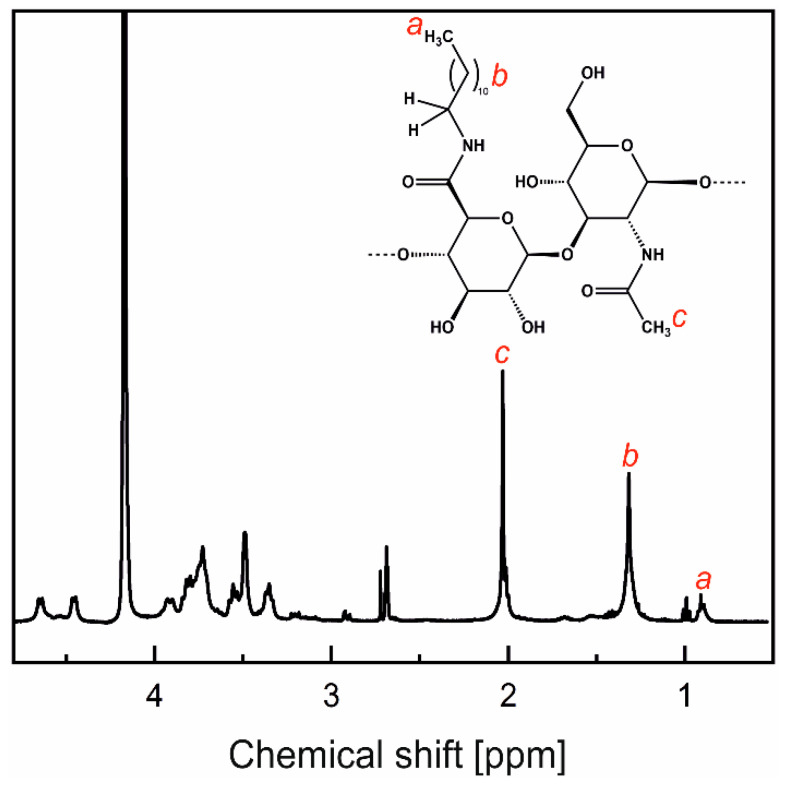
^1^NMR spectra of low-molecular-weight HA grafted with dodecyl groups (L-HA-C12) dissolved in the D_2_O/DMSO-*d*_6_ mixture (4:1, *v*/*v*) measured at 80 °C. The red lowercase letters show the assignment of protons in the chemical structure of one HA-C12 unit shown as an inset.

Hydrophobically modified H-HA and L-HA (HA-C12s) were obtained by covalent attachment of dodecylamine (C12-NH_2_) to the polysaccharide chain via an amide bond using an EDC/NHS coupling reaction [17]. First, the sodium salt of HA was converted into the acid form. To this end, an aqueous solution of sodium hyaluronate (10 mg/mL) was passed three times through a column of Dowex 50WX8-400 resin (5 mL, 1.7 milliequivalents/mL, H^+^ form; freshly washed with water/methanol/water). The polymer was recovered by lyophilization using a Beta 2-8 LSCbasic freeze dryer (Martin Christ Gefriertrocknungsanlagen, Osterode am Harz, Germany). The sample was freeze-dried at –49 °C and 0.47 mbar for 3 days. HA (200 mg) was then dissolved in formamide (40 mL) at a temperature of up to 40 °C with continuous stirring. Agitation was maintained until HA was completely dissolved. EDC (73.26 mg) and NHS (120.10 mg) were added, and the mixture was stirred for 20 min. A solution of C12-NH_2_ (68.73 mg dissolved in 40 mL DMF) was added, and the mixture was stirred at 37 °C for 24 h. After this time, the mixture was transferred to a dialysis tube and dialyzed against ethanol, a 1:1 ethanol/water mixture, and finally deionized water for three days in each of these solvents. The products were isolated by lyophilization (90–95% yield). The degree of substitution (DS) of HA with dodecyl groups was determined from NMR spectra (Figure 1), as the ratio of the peak areas of the methyl protons of the acetamide group of HA (δ = 2.0 ppm) and the protons of the methyl groups of the dodecyl substituent (δ = 0.87 ppm). The results are presented in Table 1. NMR spectra were measured using a Bruker Avance III HD 400 MHz NMR spectrometer (Bruker, Billerica, MA, USA).

### 2.9. Preparation of HA-C12/Glyceryl Trioctanoate (GTO) Nanocapsules

The nanocapsules were prepared using sonication-assisted emulsification, as described previously [15]. Briefly, GTO (10 mg) was mixed with an aqueous dispersion of H-HA or L-HA (1 mL, 1 mg/mL). The samples were vortex stirred for 5 min and sonicated for 1 min using a VCX 500 sonicator (Sonics&Materials. Inc., Newtown, CT, USA) (power—200 W, pulse duration—1 s, break duration—1 s).

## 3. Molecular Dynamics (MD) Simulations

### 3.1. Simulated Systems and Models

HA-C12 model. An oligomer consisting of 20 disaccharide units (corresponding to ca. 8 kDa) was as a model for hyaluronic acid. The HA building block was generated using the CHARMM-GUI Glycan Reader & Modeler Input Generator [18,19,20,21] and then replicated with a custom-made program to obtain a 20-unit chain. The main HA chain was subsequently substituted randomly with two C12 side groups, yielding a hydrophobically substituted oligomer with DS = 10%. Two such HA-C12 isomers were generated, one with the dodecyl groups grafted relatively close to each other (isomer A) and the other with widely spaced substituents (isomer B).

Glyceryl trioctanoate (GTO) droplet model. The geometry and topology of the GTO molecule were based on the CHARMM-GUI glycerol tripalmitate model (PPPTG in the CHARMM nomenclature), after trimming the acyl chains to the proper length. 1000 such molecules were accommodated into a 10 × 10 × 10 grid, which was energy minimized and subjected to a short 125 ps simulation in vacuo. The resulting GTO aggregate was placed in a larger simulation box and solvated with water. The energy of the system was minimized de novo and after a 1 ns equilibration, a production run of 50 ns was performed. The resulting spherical GTO droplet was used as an oil droplet model in the subsequent simulations.

HA-C12 oligomers in water. To study the behavior of the HA-C12 oligomers in an aqueous medium and to obtain their molecular models characterized by more realistic conformations, we simulated a system containing four HA-C12 oligomers (two of each isomer) immersed in an aqueous phase with an ionic strength of 0.15 M. The system was constructed by aligning 4 oligomers along the long edges of a cuboid, followed by the addition of water and the appropriate number of Na^+^ cations and Cl^–^ anions to neutralize the negative charge of HA and provide the desired ionic strength. The system was subjected to the standard simulation routine, i.e., energy minimization, short equilibration runs in the *NVT* and then *NpT* ensemble regimes, and finally a production run of 100 ns.

GTO droplet with HA-C12 oligomers (system HA-C12/GTO). To mimic the interaction of HA-C12 with the oil droplet we constructed a system containing the GTO droplet and two HA-C12 oligomers (one isomer A and one isomer B). The oligomer molecules were positioned close to the surface of the GTO droplet using the “membed” procedure implemented in the GROMACS package. The simulation box was then enlarged to avoid self-interaction of the oligomers with their periodic images; additional water molecules and ions were introduced. The simulations of the system were performed as described in the previous paragraph but with a production run of 200 ns.

### 3.2. Details of Simulations

The simulations were carried out using the GROMACS 2024 simulation package [22,23]. All molecules were described using CHARMM36 forcefield parameters generated with CHARMM-GUI. The C12 substituents in the hydrophobically modified HA oligomers were described using parameters for lipids, to ensure compatibility with the GTO parametrization. Water molecules were described with the CHARMM-recommended TIP3P(s) model. All simulations were performed at 298 K, with temperature control via either the Berendsen [24] (equilibrations runs) or Nose-Hoover [25] (production runs) thermostats. Pressure in the systems was controlled with the isotropic Berendsen (equilibration run) and Parinello–Rahman [26] (production run) barostats. The reference pressure was 1 bar, and the compressibility was 4.5 × 10^−5^ bar^−1^. All hydrogen-containing bonds were constrained using the LINCS algorithm. Dispersive interactions and short-range repulsions were described by the Lennard–Jones potential with a force-based switching cut-off of 1.2 nm. Electrostatic interactions were calculated using the particle-mesh Ewald (PME) method [27].

## 4. Results and Discussion

### 4.1. Degradation of High-Molecular-Weight Hyaluronic Acid (H-HA)

**Figure 2:**
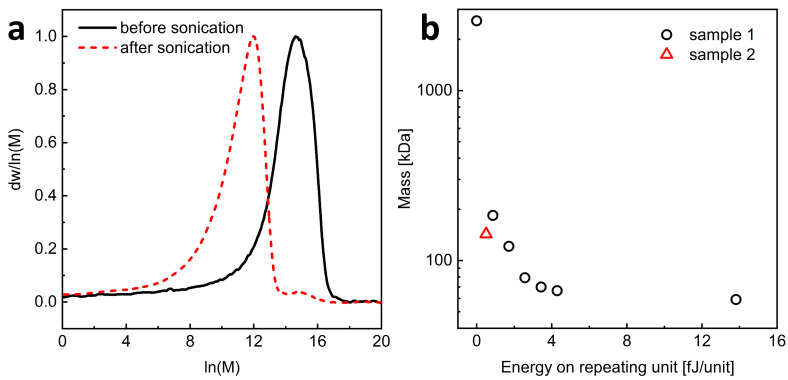
(**a**) SEC traces obtained after different times of sonication of high molecular weight (H-HA) for sample 2. (**b**) Effect of the amount of energy applied on the one repeating unit on the HA mass. Red color indicates measurements made for sample 2.

Ultrasonication was shown to be a convenient method for reducing the *M*_w_, viscosity, and molecular radius of HA without changing its chemical structure [28,29]. Therefore, we used this method to prepare L-HA from H-HA of natural origin. Commercially available H-HA with the weight-average molar mass (*M*_w_) of about 1350 kDa (determined using the PEG standards) and the molar mass dispersity (the *M*_w_/*M*_n_ ratio) of 1.46 was subjected to ultrasonic degradation, and the process was monitored by SEC measurements Figure 2 and Figure S1). We first determined the relationship between the power delivered per unit of polysaccharide and the decrease in HA molecular weight. For this purpose, the sample with a lower polysaccharide concentration (sample 1) was ultrasonicated. Figure 2 depicts that the molecular weight of the polymer decreased rapidly with sonication time to a value of about 50 kDa, and further sonication of the sample did not lead to HA degradation. Remarkably, the molar mass dispersity increased slightly during degradation (Table S1). This indicates that our sonication conditions were sufficient to break the polysaccharide chains, but the process leads to a certain constant value of polysaccharide length (Figure 2 and Table S1). The effects of sonication intensity, temperature, HA concentration, and ionic strength on ultrasonic degradation of HA were studied previously [30]. It was shown that mechanical degradation, which is the sonication process, always leads to a constant value of the molecular weight of the degraded HA. It is considered to correspond to the critical molecular length that can diffuse the loaded mechanical stress without breaking covalent bonds. The values of this limiting *M*_w_ were almost equal regardless of the HA origin, the polymer concentration, and the nature of co-existing cations under the same ultrasonic intensity.

From the mass–energy relationship (Figure 2b), it can be read that to obtain HA with a molecular weight close to 200 kDa, it is necessary to deliver an energy of about 0.5 × 10^−15^ J per HA unit. Then, the degradation process was repeated for a larger amount of H-HA (sample 2), applying the energy of 0.5 × 10^−15^ J per saccharide unit. The result was an HA material with *M*_w_ of ca. 110 kDa and the *M*_w_*/M*_n_ ratio equal to 1.69 (Table S1). A smaller peak with a maximum at ln(M) of about 15 can be observed, indicating the presence of trace amounts of non-degraded polysaccharides after the sonication process.

### 4.2. Synthesis of Amphiphilic HAs (AmHAs)

The dodecyl groups were attached to the polysaccharide chains via amide bounds, as a result of the reaction between the carboxyl groups of HA and dodecylamine. NMR spectroscopy was used to confirm the composition of the products. As an example, the NMR spectrum of L-HA-C12 is shown in Figure 1. The signals at approximately 1.3 and 0.9 ppm correspond to the protons of the methylene (CH_2_) groups and the terminal methyl group of the dodecyl moieties, respectively, while the peak at ca. 2.0 ppm was assigned to the methyl protons of the HA acetamide group. DS with the dodecyl groups was calculated based on the ratio of the peak areas for the methyl groups of the dodecyl substituent and the acetamide group of HA (Table 1).

The HA derivatives grafted with dodecyl groups were previously prepared using various synthesis procedures. For example, Szafraniec et al. performed the reaction of HA sodium salt with C12-NH_2_ using EDC/NHS coupling in a heterogeneous water/DMF/chloroform mixture [10]. This mixture was proposed since the amine is not soluble in water. On the other hand, Cadete et al. reported the preparation of dodecylamide-functionalized HA by reacting tetrabutylammonium salt of HA with dodecylamine using 2-bromo-1-ethyl pyridinium tetrafluoroborate as the amide coupling reagent [5]. In this case, the reaction medium was a DMF/monomethyl formamide mixture, which provided good solubility for all reactants (homogeneous conditions). However, the procedure required the conversion of HA sodium salt to tetrabutylammonium hyaluronate before the reaction with the amine and the removal of these cations after the reaction. The synthesis route we used requires only the conversion of sodium hyaluronate into its acid form, which is soluble in a DMF/NMF mixture, as is dodecylamine, so the coupling reaction proceeds under homogeneous conditions.

### 4.3. Measurements of Critical Aggregation Concentration (CAC)

**Figure 3:**
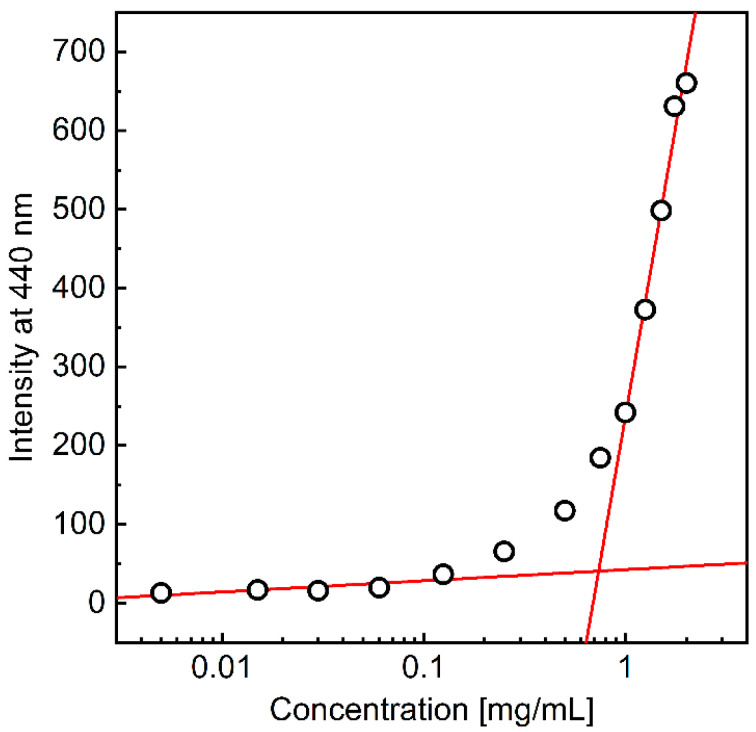
The fluorescence intensity of DPH (4 μM) at 440 nm (λ_exc_ = 375 nm) as a function of the L-HA-C12 concentrations.

Hydrophobically modified polysaccharides are expected to self-organize into various structures when dispersed in an aqueous environment above the critical aggregation concentration (CAC) [31]. To determine the CAC for the AmHAs, a series of samples containing a constant concentration of DPH, a fluorescence probe, and varying polymer content were prepared, and the fluorescence intensity of DPH was measured (Figure 3). DPH, as a hydrophobic compound, aggregates in aqueous environments, leading to the disappearance of fluorescence in these environments. The fluorescence intensity of the probe increases significantly in hydrophobic environments. Thus, the observed rise in DPH fluorescence intensity with increasing polymer concentration in the system is associated with the penetration of the fluorophore molecules into the hydrophobic domains formed by the HA-C12 alkyl groups once the CAC is exceeded. The CAC value was determined as the intersection of two lines fitted to regions defined for low polymer concentrations and when the DPH fluorescence intensity increased rapidly (Figure 3). Table 1 shows the determined CACs.

The aggregation processes of various hydrophobic HA derivatives were previously investigated, and CAC values were determined. Generally, the self-assembly process is influenced by several factors related to the polymer itself, such as the degree of substitution (the most important parameter) [32], the type of hydrophobic moiety, as well as the environment in which it is suspended (including ionic strength and pH) [33,34]. For example, Eenschooten et al. showed that the CAC of octenyl succinate-grafted HA decreases linearly with increasing the degree of modification of the polymer (with DS between 6 and 43%) [29]. Higher hydrophobicity of the derivatives results in aggregation at a lower concentration. Aggregation of oleyl-modified HA (DS of 6.2%) in water and PBS was previously studied with surface tension and fluorescence measurements [30]. The CAC of this polymer was approximately 30 and 60 µg/mL in PBS and water, respectively, indicating that the ionic strength of the environment significantly affects the self-organization process of AmHAs.

The polymer concentration values corresponding to the formation of hydrophobic domains determined in this study are comparable to those previously reported for other alkylated HA derivatives. It should be emphasized that the CAC value strongly depends on the length of the alkyl chain attached to the polysaccharide. For example, Velcer et al. showed that the CAC decreased with increasing alkyl chain length and with increasing hyaluronan molecular weight at a comparable DS [35]. HA modified with alkyl chains containing 6 carbon atoms and 16 carbon atoms created polymer micelles at concentrations of 5040 and 60 µg/mL, respectively. Similarly, Pelletier et al. demonstrated that HA modified with alkyl chains containing 12 carbon atoms (DS of 5%) and 18 carbon atoms (DS of 2%) formed hydrophobic domains at polymer concentrations of 50 µg/mL and 10 µg/mL, respectively [36]. Although direct comparison of these findings with those of our work is challenging due to differences in initial HA molecular weight, nature of the hydrophobic pendant groups, and DS, the order of magnitude of the HA concentrations at which aggregation occurred indicates a relatively good agreement with the literature. Both our results and the values reported in the literature indicate that AmHAs aggregate spontaneously at very low concentrations.

### 4.4. TEM Visualization

**Figure 4:**
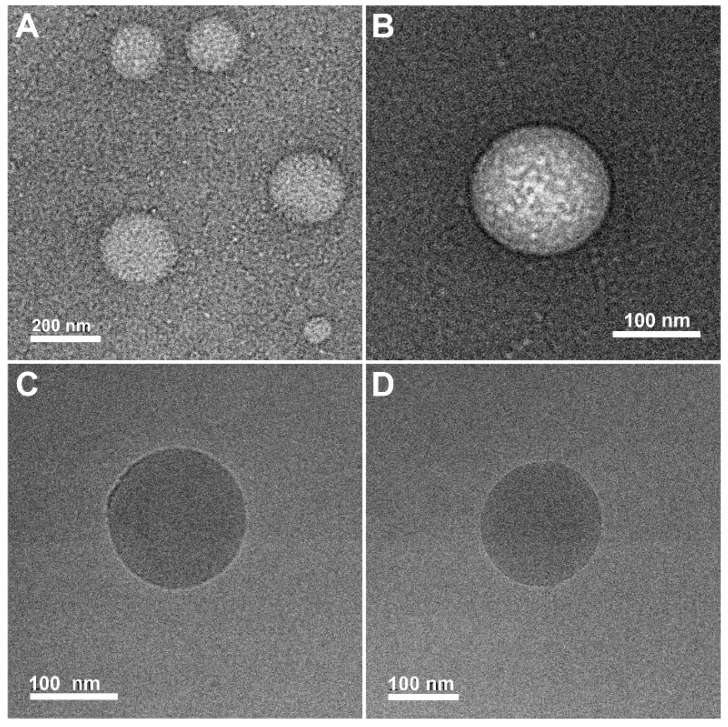
Typical TEM micrographs of the objects formed in the dispersion of H-HA-C12 (**A**) and L-HA-C12 (**B**). Both samples (2 mg/mL concentration) were stained with UranyLess. Typical cryo-TEM micrographs of nanocapsules covered with H-HA-C12 (**C**) and L-HA-C12 (**D**).

To study the morphology of the objects formed in the dispersion of AmHAs above the CAC, we attempted to use cryo-TEM microscopy, which allows direct observation of the sample without drying it. L-HA-C12 and H-HA-C12 samples suspended in PBS at a concentration of 2 mg/mL were visualized under the cryo-TEM microscope. Unfortunately, the use of this method failed to obtain images of the objects. This may be due to the strong hydration of the polysaccharide backbone. Using molecular dynamics simulations, it was shown that more than 20 water molecules are attached to each disaccharide unit via hydrogen bonds in the first hydration shell [37]. In addition, the short alkyl chains used to modify the HA chains result in weak intramolecular interactions; therefore, H-HA-C12 and L-HA-C12 polymers aggregate primarily as a result of intermolecular interactions between alkyl groups attached to different chains. This results in the formation of loosely packed and highly hydrated structures with a wide size distribution, giving low contrast in cryo-TEM observations. Therefore, the morphology of the structures formed by the HA derivatives was visualized in the dry state using conventional TEM microscopy with negative staining (Figure 4). For both polymers, the particles were approximately spherical in shape but differed in size. In the case of L-HA-C12, the diameters of the objects were in the range of 50–150 nm, while the H-HA-C12 particles were characterized by a wider size distribution, with diameters between 40 and 500 nm. It should be noted that the drying process results in a significant reduction in particle sizes and can lead to substantial changes in their morphology.

### 4.5. Preparation of Oil-Filled Nanocapsules (NCs)

The oil-core nanostructures were prepared by simply mixing GTO with the polymer solution (H-HA/C12 or L-HA/C12) at a volume ratio of oil/water phase of about 1:100, followed by sonication. Both as-prepared dispersions were characterized by an opaque, milky color, much more intense than dispersions of the polymer alone. We used DLS (Table 1) and cryo-TEM microscopy (Figure 4) to measure the size of NCs and visualize their morphology. The microphotographs (taken one week after their preparation) reveal the presence of spherical objects with good contrast and sharp well-defined boundaries. The estimated sizes based on the microscopic images were about 210 and 130 nm for L-HA-C12/GTO and H-HA-C12/GTO, respectively, which roughly corresponds to the sizes obtained by the DLS method.

### 4.6. NC Stability

**Figure 5:**
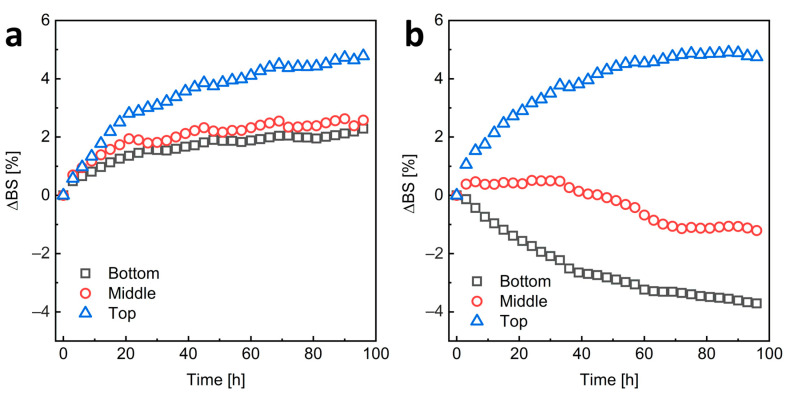
The mean changes in light backscattered by the sample over time in the lower, middle, and upper parts of the L-HA-C12/GTO (**a**) and H-HA-C12/GTO (**b**) nanoemulsions.

Due to the very low transparency of the samples, we assessed the stability of HA-C12-coated NCs by measuring the intensity of backscattered light. To study the migration of nanoparticles and changes in their size over time, the measurements were performed at the bottom, middle, and top of the sample tube over a period of 96 h (4 days) (Figure 5). For H-HA-C12/GTO, a decrease in scattering at the bottom of the sample tube due to a decrease in the concentration of particles in this part and an increase at the top part of the sample due to an increase in the concentration of the dispersed phase and possible an increase in object size due to coalescence or flocculation phenomena were observed. This suggests upward migration of NCs, namely creaming. Creaming is a common phenomenon of instability in emulsions or suspensions when the dispersed phase has a lower density than the continuous phase. The L-HA-C12/GTO nanoemulsion seems to be more stable. In this case, there is a slight increase in backscattering in all areas of the sample, indicating an increase in object size due to coalescence or flocculation phenomena. However, the changes in scattering are not large (the increase in backscattered light is less than 5%) and stabilize after about 60 h. The greater stability of NCs coated with the shorter HA-C12 is also indicated by the zeta potential results (Table 1). The ζ value for system L-HA-C12/GTO is close to −30 mV, the value at which the particles are considered stable). The H-HA-C12/GTO nanocapsules H-HA-C12/GTO are characterized by a much lower value, which may affect their lower stability.

### 4.7. The Interaction between HA-C12 and GTO Nanodroplet

**Figure 6:**
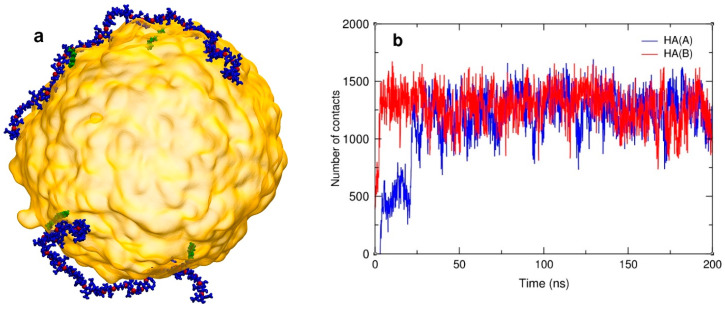
A snapshot of the HA-C12/GTO system was taken at the end of the simulation (200 ns) (**a**). The GTO droplet is depicted in yellow. HA main chains and C12 groups are shown in dark blue and green, respectively. Water and ions are not shown for clarity. Number of contacts between the dodecyl side groups and the GTO molecules over time (**b**).

To gain some insight into the behavior of HA-C12 at the water–oil interface, we performed several MD simulations. The simulated system consisted of the GTO droplet (1000 molecules of triglyceride) and two HA-C12 oligomers. The final configuration of the system is shown in Figure 6a. Visual analysis of the trajectory shows that both HA-C12 oligomers remained in close contact with the GTO droplet throughout the whole simulation period. This behavior of HA-C12 chains is due to the penetration of the dodecyl side groups deeper into the hydrophobic interior of the GTO droplet, which can be demonstrated by calculating the number of individual contacts between the dodecyl atoms and the GTO atoms. Figure 6b confirms a fast increase in such contacts number within the first 25–30 ns, after which this number remains constant until the end of the simulation. To establish possible interactions of GTO with the oligomer main chain, a radial distribution function (RDF) was calculated for the oxygen atoms of the polar sites of HA (hydroxyl, carboxyl, and carbonyl in the amide bond) and the glycerol part of GTO (ester OSL and carbonyl OBL). These atomic pairs are likely to be involved in an intermolecular interaction, for instance via a hydrogen bond or a water bridge. The RDF plot for all these pairs (Figure S3) shows that some hydroxyl groups of HA are directly hydrogen bonded to GTO carbonyl groups, as indicated by the presence of a marked maximum at 0.28 nm. However, the average cumulative number of OBL atoms found in the first coordination sphere of OH groups is merely 0.04, which means that each oligomer forms on average only 3–4 -OH×××O=C- hydrogen bonds to the GTO molecules. Some other RDFs show less prominent maxima, located in the range of 0.42–0.45 nm. These values correlate well with a distance of two carbonyl oxygen atoms (actually, any two oxygen atoms) linked together via a water bridge (Figure S3), so this maximum may be attributed to this interaction type. The number of OBL and OSL atoms found in the second coordination sphere of the hydroxyl and amide O atoms equals ca. 30 per oligomer (i.e., ca. 1.5 per disaccharide unit). Thus, it may be concluded that although the driving force for the deposition of HA-C12 on the surface of the GTO nanodroplets is the Lennard–Jones attraction between the hydrophobic parts of HA-C12 and the oil droplet, the aggregate gains additional thermodynamic stability due to the linking of the HA main chain to the glycerol site of GTO either via direct hydrogen bonds or water bridges.

### 4.8. Impact of the GTO-HA-C12 Interaction on the Hydration and Flexibility of the HA Chain

**Figure 7:**
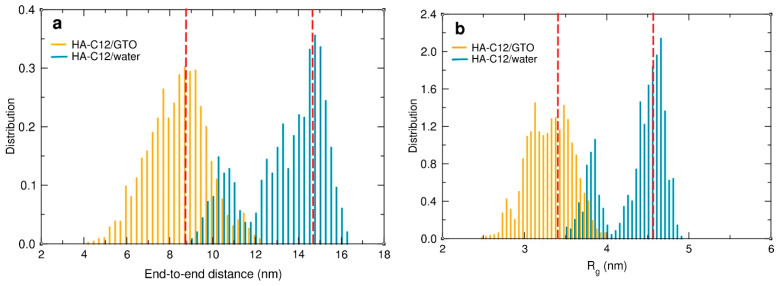
Distributions of the end-to-end distances (**a**) and radius of gyration (**b**) of the HA-C12 oligomers interacting with the GTO droplet (orange) and with water (blue) were calculated from the simulations. The red dashed vertical lines correspond to the average values. The distributions were averaged over the last 100 and 50 ns of the trajectories for HA-C12/GTO and HA-C12/water systems, respectively, and over all the oligomer molecules present in the systems.

The anchoring of the HA chain to the surface of the GTO nanodroplet can be expected to adversely affect its flexibility and hydration. To quantify these effects, we calculated the end-to-end distance (*D*_ee_), the radius of gyration (*R*_g_), and the number of hydrogen bonds (H-bonds) with water molecules for both oligomers interacting with the GTO droplet and compared the results with the values calculated for HA-C12 in water. The time evolution of both *D*_ee_ and *R*_g_ in the HA-C12/GTO system (Figures S4 and S5) follows a similar pattern. Large changes can be observed within the first 100 ns of the simulation, after which the values practically stabilize. This reflects the process of adaptation of the conformation of the polysaccharide chains to the arrangement imposed by immersing the hydrocarbon groups into the GTO medium. Once the strains have been released and the chains found a local conformational minimum, they tend to maintain these optimal conformations. The distributions of both quantities averaged over the last 100 ns are shown in Figure 7. The polysaccharide chains interacting with the oil droplets are coiled, but they can vary their conformations fairly freely, as evidenced by the wide distribution of *D*_ee_. The four HA-C12 oligomers simulated in an aqueous medium behave quite differently. Since there were no constraints on their movement, the main chains stretched freely (Figure S2) due to repulsion between the negatively charged groups located along the HA chain, as reflected in the increase in both the *D*_ee_ and *R*_g_ values (Figure 7).

**Figure 8:**
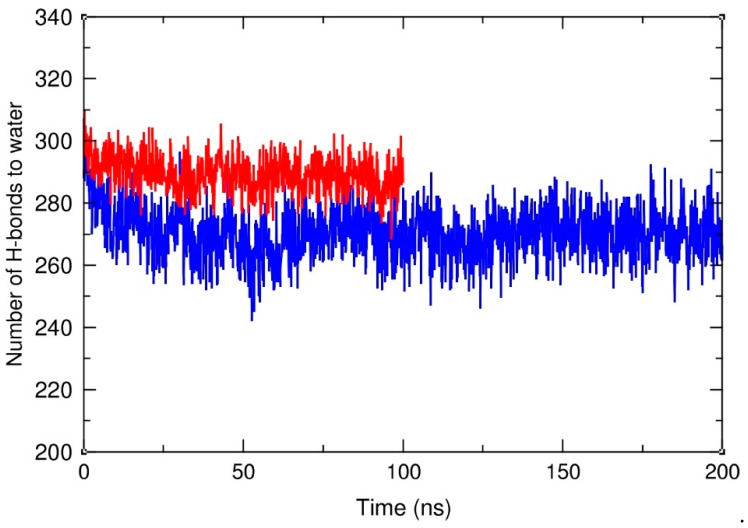
Number of hydrogen bonds to water molecules calculated per one HA chain in water (red) and in the HA-C12/GTO system (blue).

The close contact of HA-C12 with the hydrophobic surface of the GTO droplet may also affect the degree of hydration of the HA polysaccharide chain. To clarify this issue, we counted the hydrogen bonds formed between the HA-C12 oligomer and water molecules in the HA-C12/water and HA-C12/GTO systems. Standard criteria for the presence of H-bonds were applied, i.e., the maximum accepted length of the hydrogen bridge was set to 0.35 nm and the maximum allowed deviation from linearity was set to 30 degrees. Figure 8 clearly shows that the hydration state of HA-C12 decreases at the initial stage of the simulations due to the deposition of the polymer on the hydrophobic surface of the droplet. After about 100 ns, it stabilizes around the value of 270 ± 8 H-bonds per oligomer. The average number of H-bonds per oligomer in an aqueous environment, calculated for the last 50 ns, equals 298 ± 7. This shows that the degree of hydration decreases only by about 9% due to the binding of the oligomer to the nanodroplet surface.

## 5. Conclusions

In this work, we focused on the application of ultrasounds in the preparation of oil-core nanocapsules coated with AmHAs. Special attention was paid to the effect of polysaccharide chain length on the properties of the obtained nanostructures. The results indicate that sonication is a very convenient method that allows for a controlled reduction in HA molecular weight. Importantly, the molecular weight dispersion of the polysaccharide material increases only slightly after ultrasonic degradation. In addition, HA can be effectively grafted with alkyl groups by converting sodium hyaluronate to its acid form, which is soluble in DMF/NMF mixture, similar to alkylamines, thus avoiding the use of heterogeneous conditions during the reaction. Due to intermolecular interactions between alkyl groups attached to different chains, HA-C12s with DS of about 17% self-organize into loosely packed and highly hydrated nanogels with a wide size distribution when dissolved in water at concentrations higher than a few tens of µg/mL. Ultrasound-assisted emulsification of an appropriate hydrophobic phase in the aqueous solutions of these polysaccharides can yield nanocapsules with an oil core stabilized by the presence of HA-C12s. Whereby, L-HA-C12-coated NCs exhibited greater stability compared to the polysaccharide with the longer chain.

This study also provided molecular insights into the conformation and hydration of the HA main chain on the surface of oil nanodroplets. The simulations showed that HA-C12 readily adsorbs at the water–oil interphase adopting a more compact conformation compared to that in the aqueous phase. The hydrophobic groups are immersed in the oil droplet, while the main polysaccharide chain remains in the aqueous phase forming a corona on the surface of the nanodroplet. The important result of our simulations is that the HA hydroxyl groups interact with the polar part of the triglycerides via direct hydrogen bonds or water bridges. As a result, the hydration number of HA-C12 decreases slightly, but the stability of the formed nanocapsules with an oil core increases.

In conclusion, ultrasound-assisted preparation of nanocapsules does not require the use of organic solvents and surfactants, which is of great importance in the formulation of drugs, foods, and nutraceutical supplements. Further research should focus on evaluating the efficiency of encapsulation and distribution of bioactive compounds in NC oil cores.

## Figures and Tables

**Table 1:** Values of the degree of substitution (DS) and critical aggregation concentration (CAC) for the HA derivatives. Values of the mean hydrodynamic diameter (*D*_h_). Polydispersity (PDI) and zeta potential (ζ) of HA-C12s dispersed in a 1 mM NaCl solution of pH 7.4 *^a^.*

Polymer	DS [%]	CAC [µg/mL] (*n* = 2)	*D*_h_ [nm]	PDI	ζ [mV] (*n* = 3)
L-HA-C12	17.4	73.5 ± 3.2	-	-	−27.4 ± 0.7
H-HA-C12	17.0	79.2 ± 3.9	-	-	−16.1 ± 0.5
L-HA-C12/GTO	-	-	244	0.38	−26.5 ± 0.4
H-HA-C12/GTO	-	-	165	0.52	−3.6 ± 0.45

*^a^* Values are the mean ± standard deviation of *n* measurements.

## Data Availability

Data are contained within the article and the Supplementary Materials.

## Supplementary Materials

The following supporting information can be downloaded at https://www.mdpi.com/article/10.3390/ma17184524/s1. Figure S1. SEC traces for the HA material obtained after different time of sonication of sample 1. Figure S2. Snapshots of the system of four HA-C12 oligomers in the aqueous medium at 0 ps (a) and 100 ns (b). Both isomers, A and B, are depicted in red and green, respectively. Water and ions are not shown for clarity. Figure S3. RDF functions for the oxygen atoms of the HA main chain (hydroxyl, carboxyl and amide carbonyl) and the oxygen atoms of the GTO glycerol part: ester (OSL) and carbonyl (OBL). The sketch on the right illustrates the geometry of two carbonyl oxygen atoms interacting via a water bridge. The distance 0.27 nm corresponds to the location of the first maximum in the RDF of the carbonyl oxygen – water oxygen pair. Figure S4. Time evolution of the end-to-end distance (Dee) in the HA-C12/TOG system (a) and the HA-C12/water system (b). Figure S5. Time evolution of the radius of gyration (Rg) in the HA-C12/TOG system (a) and the HA-C12/water system (b). Table S1. The values of weight-average molecular weight (*M*w), number-average molecular weight (*M*n), and dispersity index (*M*w/*M*n) of HA after different time of sonication**.**
