# Supplementary material for: Ultrasound-Assisted Preparation of Hyaluronic Acid-Based Nanocapsules with an Oil Core
Source: Materials (Basel). 2024 Sep 14;17(18):4524. doi: 10.3390/ma17184524 (PMC11433493; doi:10.3390/ma17184524)
Supplement: Supplementary file 1 [file materials-17-04524-s001.zip › materials-3186914-supplementary.pdf]

Supplementary Materials for

Ultrasound-assisted preparation of hyaluronic acid-based nanocapsules with an oil core

Natan Rajtar[1,2], Grzegorz Łazarski[1,2], Aleksander Foryś[3], Barbara Trzebicka[3], Dorota Jamróz[1], Mariusz Kepczynski*,[1]

[1] Faculty of Chemistry, Jagiellonian University, Gronostajowa 2, Kraków 30-386, Poland

[2] Doctoral School of Exact and Natural Sciences, Jagiellonian University, Prof. S. Łojasiewicza 11, 30-348 Krakow, Poland

[3] Centre of Polymer and Carbon Materials, Polish Academy of Sciences, Zabrze 41-819, Poland

Degradation of HA. Commercially available H-HA of about 1350 kDa was degraded by sonication, and the process was monitored using SEC measurements (**Figure S1**). For sample 1, it was found that the molecular weight of the polymer material decreased gradually with sonication time.

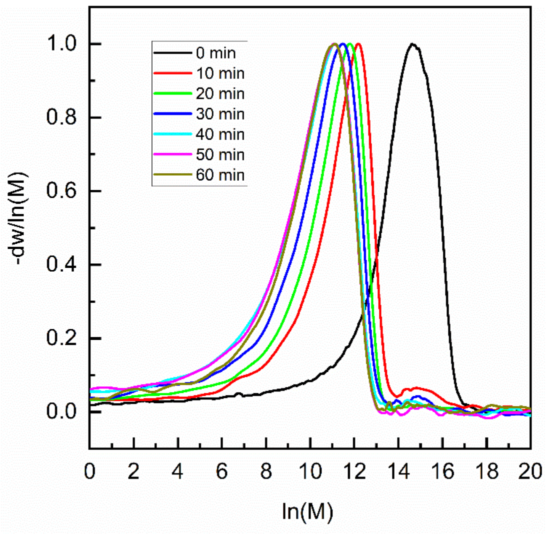

Figure S1. SEC traces for the HA material obtained after different time of sonication of sample 1.

For each polymer, the weight-average molecular weight (M_w) and number-average molecular weight (M_n) were calculated using the following equations:

$$M_n = \Sigma h_i M_i \quad (1)$$

$$M_w = \frac{\Sigma h_i (M_i)^2}{M_n} \quad (2)$$

where: h_i is the number of polymer chains with molecular weight of M_i. The mass at peak maximum (M_p) read from the graph is also shown in **Table S1**.

Table S1. The values of weight-average molecular weight (M_w), number-average molecular weight (M_n), and dispersity index (M_w/M_n) of HA after different time of sonication.

Sonication time	Energy	M_n [kDa]	M_w [kDa]	M_w/M_n	M_p [kDa]
Sample 1					
0 min		916	1334	1.46	2239
10 min		94	151	1.61	194
20 min		51	86	1.69	132
30 min		38	68	1.79	95
40 min		22	48	2.18	67
50 min		19	40	2.11	66
60 min		27	46	1.70	68
Sample 2					
60 min		65	110	1.69	159

MD Simulations

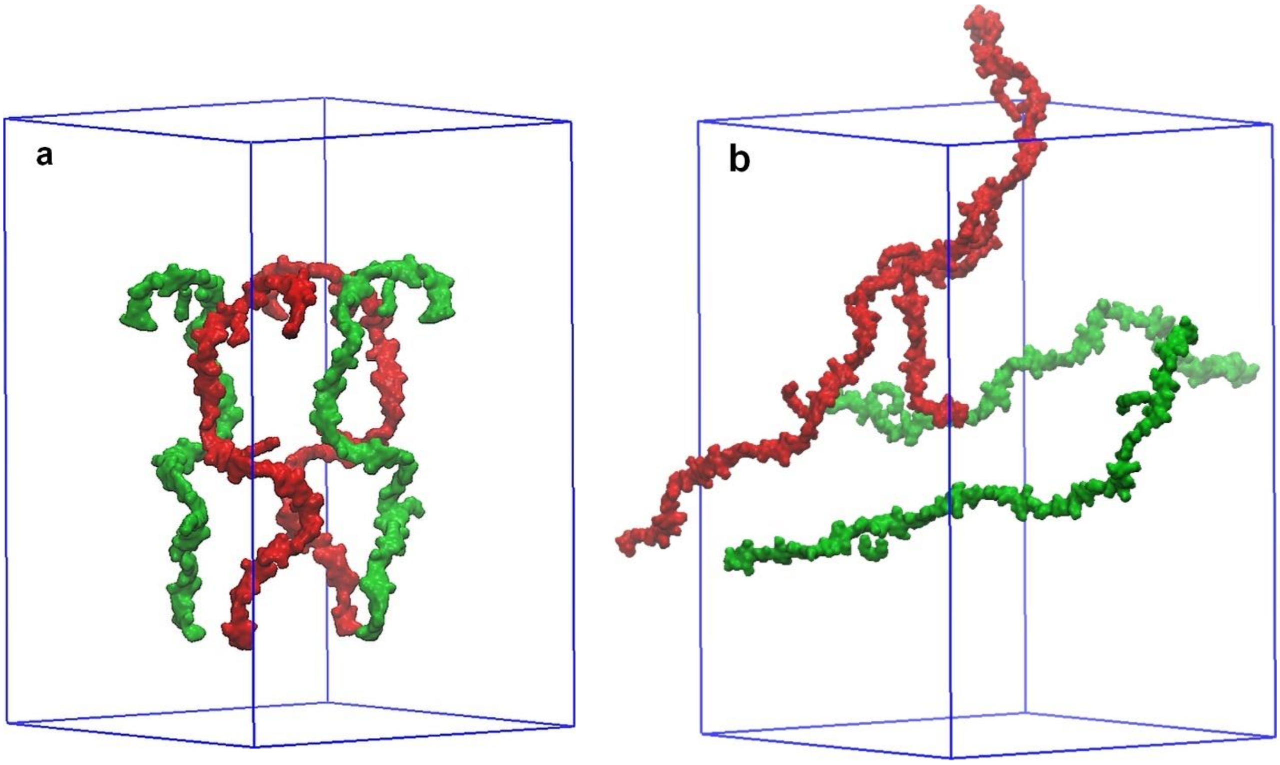

Figure S2. Snapshots of the system of four HA-C12 oligomers in the aqueous medium at 0 ps (a) and 100 ns (b). Both isomers, A and B, are depicted in red and green, respectively. Water and ions are not shown for clarity.

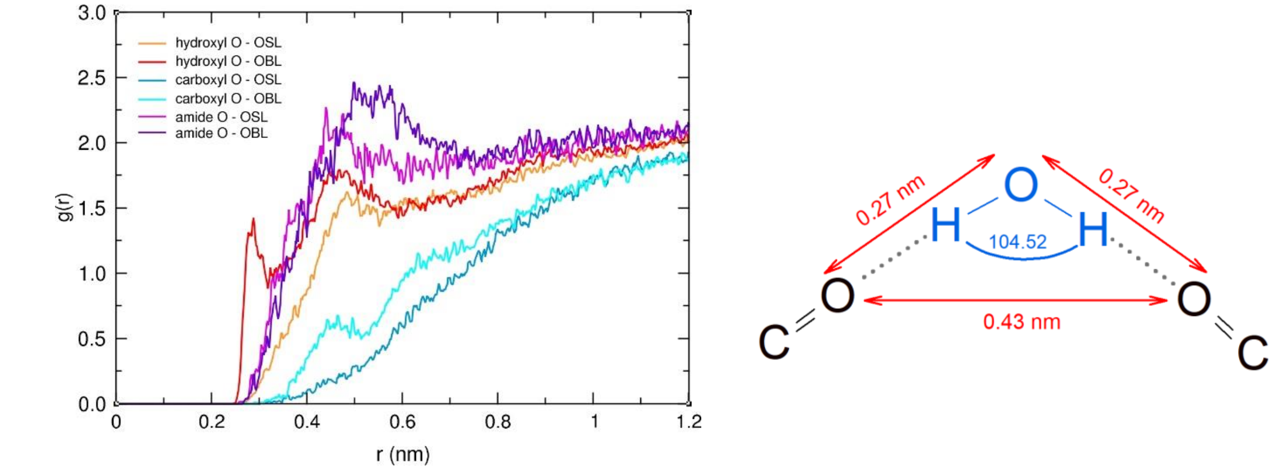

Figure S3. RDF functions for the oxygen atoms of the HA main chain (hydroxyl, carboxyl and amide carbonyl) and the oxygen atoms of the GTO glycerol part: ester (OSL) and carbonyl (OBL). The sketch on the right illustrates the geometry of two carbonyl oxygen atoms interacting via a water bridge. The distance 0.27 nm corresponds to the location of the first maximum in the RDF of the carbonyl oxygen – water oxygen pair.

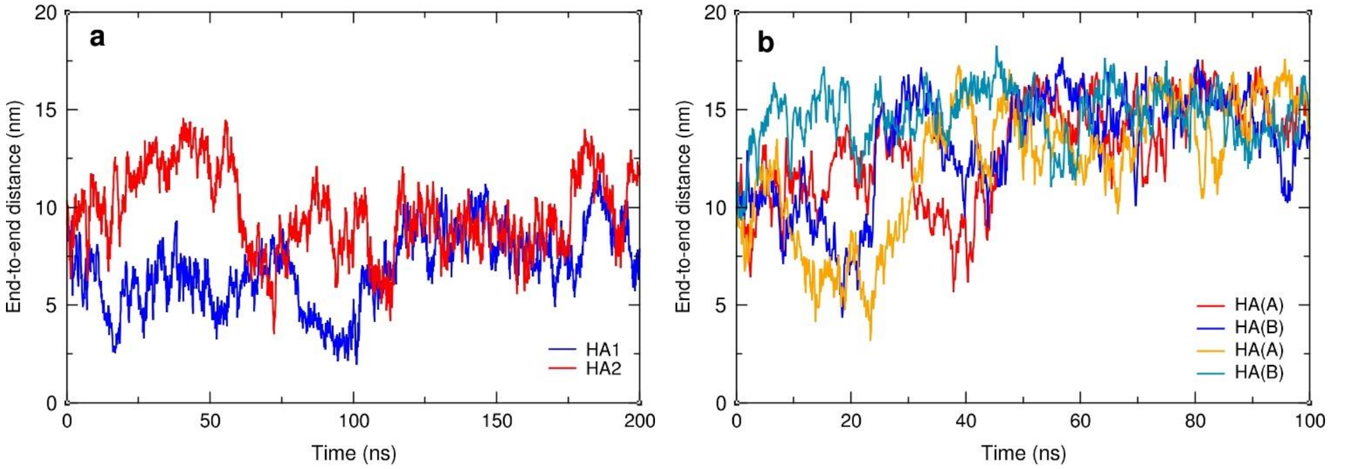

Figure S4. Time evolution of the end-to-end distance (D_{ee}) in the HA-C12/TOG system (a) and the HA-C12/water system (b).

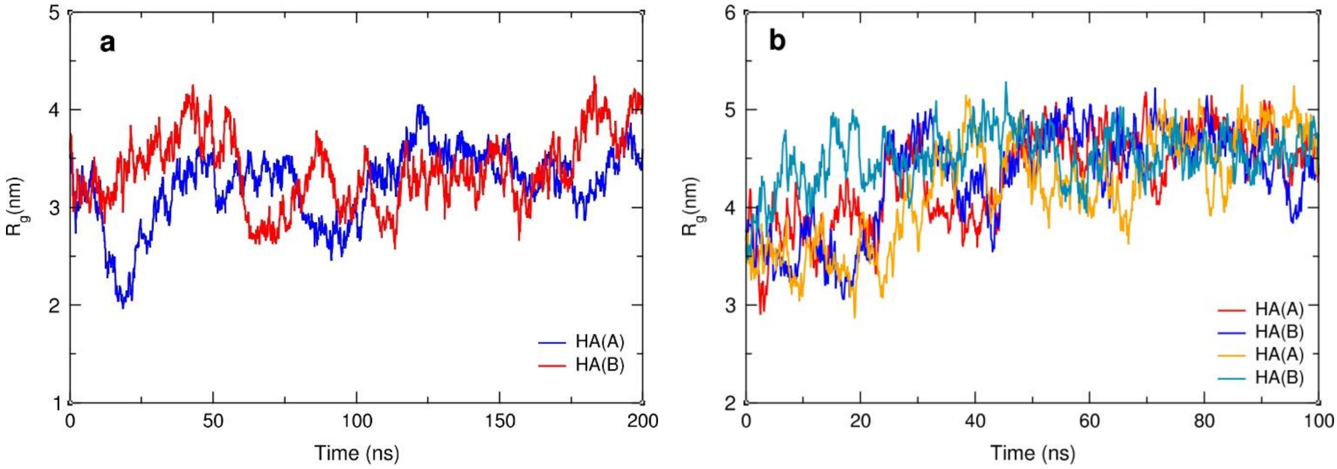

Figure S5. Time evolution of the radius of gyration (R_g) in the HA-C12/TOG system (a) and the HA-C12/water system (b).

Acknowledgments

This work was supported by the National Science Centre, Poland (grant no. 2019/35/B/ST5/02147). We gratefully acknowledge Polish high-performance computing infrastructure PLGrid (HPC Center: ACK Cyfronet AGH) for providing computer facilities and support within computational grant no. PLG/2024/017280.
